# Magnetic-field-induced insulator–metal transition in W-doped VO_2_ at 500 T

**DOI:** 10.1038/s41467-020-17416-w

**Published:** 2020-07-17

**Authors:** Yasuhiro H. Matsuda, Daisuke Nakamura, Akihiko Ikeda, Shojiro Takeyama, Yuki Suga, Hayato Nakahara, Yuji Muraoka

**Affiliations:** 1 The Institute for Solid State Physics, The University of Tokyo, 5-1-5 Kashiwa, Chiba, 277-8581 Japan; 2 Graduate School of Natural Science and Technology, Okayama University, 3-1-1 Tsushima-naka, Tsushima, Kita-ku, Okayama, 700-8530 Japan; 3 Research Institute for Interdisciplinary Science, Okayama University, 3-1-1 Tsushima-naka, Tsushima, Kita-ku, Okayama, 700-8530 Japan

**Keywords:** Electronic properties and materials, Magnetic properties and materials

## Abstract

Metal–insulator (MI) transitions in correlated electron systems have long been a central and controversial issue in material science. Vanadium dioxide (VO_2_) exhibits a first-order MI transition at 340 K. For more than half a century, it has been debated whether electron correlation or the structural instability due to dimerised V ions is the more essential driving force behind this MI transition. Here, we show that an ultrahigh magnetic field of 500 T renders the insulator phase of tungsten (W)-doped VO_2_ metallic. The spin Zeeman effect on the *d* electrons of the V ions dissociates the dimers in the insulating phase, resulting in the delocalisation of electrons. As the Mott–Hubbard gap essentially does not depend on the spin degree of freedom, the structural instability is likely to be the more essential driving force behind the MI transition.

## Abstract (web summary)

Vanadium dioxide exhibits a metal-insulator (MI) transition. The mechanism driving this behaviour has long been a subject of debate. Here, the authors show that the MI transition in heavily doped vanadium dioxide can be driven by a magnetic field, suggesting the MI transition is due to structural instability.

## Introduction

The Mott–Hubbard insulator is a class of materials in which strong electron correlation disturbs the motion of electrons and electrons are localised^1^. The magnetic ground state of many Mott–Hubbard insulators shows antiferromagnetic order, and high-*T*_*c*_ superconductivity occurs near the magnetic-order phase, indicating that the spin degree of freedom is important to understand their peculiar electronic states. On the other hand, several insulators possess strong electron correlation, but their magnetic ground state is a spin singlet (nonmagnetic)^1^. VO_2_ is one such material. Strong electron correlation has been claimed to be necessary for understanding the large energy gap of 0.7 eV in the low-temperature insulating phase of VO_2_^2–4^. A key feature of the MI transition of VO_2_ is that it occurs along with a structural transition from a high-temperature rutile (tetragonal) phase to a low-temperature monoclinic phase^5–7^. Vanadium dimers are formed in the low-temperature monoclinic phase, and *d* electrons of the V ions (V^4+^: *d*^1^) form metal–metal bonding with a molecular orbital. The *d* electrons are localised in the molecular orbital of the V–V dimer, which can result in the insulating nature^6–9^.

Many theoretical and experimental studies have been conducted to determine whether the structural instability due to dimerisation^6–9^ or electron correlation (Mott physics)^2–4,10,11^ is the more essential driving force behind the MI transition. An understanding of the microscopic mechanism of the MI transition of VO_2_ is also important for its practical applications as sensors and switching devices; abrupt changes in resistivity and optical absorption at the transition temperature 340 K are useful for them^12^. The manipulation of electron spins by a magnetic field can shed light on the problem of MI transition because the dimerisation can be suppressed by the Zeeman energy in magnetic fields. One may envision that the molecular orbital between the V atoms collapses by the forced alignment of the spin direction because the bonding orbital is formed only with two electrons having opposite spin states. The advantage of utilising a magnetic field is that the electronic state can be modified through Zeeman splitting while maintaining the quantum-mechanical electron correlation that is significant at low temperatures. Since the Mott–Hubbard gap is expected to be insensitive to the spin state, the magnetic field cannot affect the insulating nature if the Mott physics is the more essential driving force behind the MI transition. Ultra-high magnetic fields with Zeeman energy at least comparable with the thermal energy at the MI transition temperature (*T*_MI_) would be required to investigate the potential magnetic-field-induced metallisation of VO_2_.

In this study, we experimentally demonstrate that the insulating dimerised state can be transformed to a metallic state by a strong magnetic field of 500 T in W-doped VO_2_, the MI transition temperature of which is controlled to ~100 K^13^. We performed magneto-transmission experiments using a near-infrared laser line and found a significant decrease in the transmitted light intensity at the ultra-high magnetic-field region, which is distinct evidence of the field-induced insulator–metal (IM) transition. The observed onset field of the transition at 14 K is ~120 T, and its Zeeman energy corresponds to 162 K when *g* = 2 and *S* = 1/2, where *g* and *S* are the *g*-factor and spin quantum number, respectively. The observed magnetic-field-induced metallisation indicates that dimerisation is a more essential driving force than the electron correlation for the MI transition in VO_2_.

## Results

### V_1−*x*_W_*x*_O_2_ (*x* = 0.06)

**Fig. 1:**
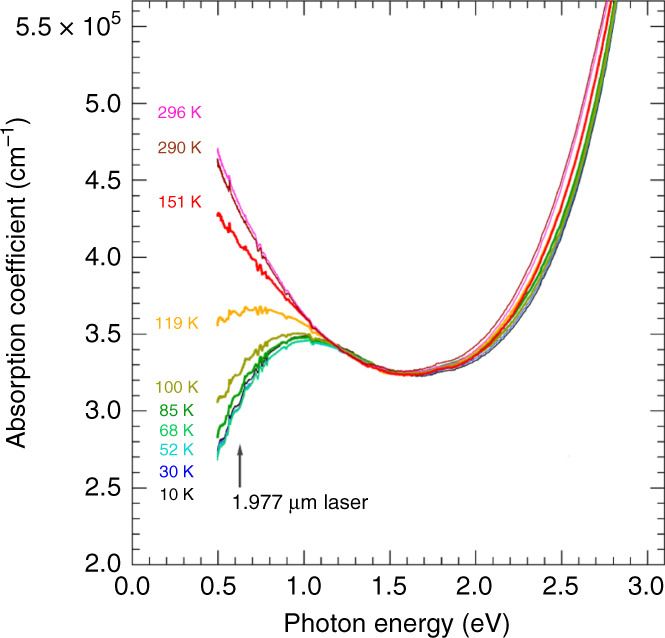
Optical absorption spectra at different temperatures without external magnetic fields. The arrow indicates the energy of the laser used for the magneto-transmission experiments.

  Figure 1 shows optical absorption spectra of a V_1−*x*_W_*x*_O_2_ (*x* = 0.06) thin film at different temperatures without external magnetic fields. The broad absorption peak at around 1 eV can be attributed to the excitation from the bonding *d*_∣∣_ orbital to the non-bonding π^*^ orbital of the *d* electrons of V^4+^ (*d*^1^) in the octahedron of oxygen ions^6,14–16^. The *d*_∣∣_ and π^*^ orbitals originate from the *t*_2*g*_ state in the crystal field with cubic symmetry. The strong increase in absorption with photon energy increasing beyond ~1.8 eV is attributed to the transition of charge transfer from the vanadium 3*d*-like band to the oxygen 2*p*-like band^15^.

The absorption below ~1.2 eV is found to increase with decreasing photon energy at a high temperature of, for example, 296 K. The absorption becomes less significant as the temperature decreases. This behaviour is accounted for by the opening of the energy gap and a change in the number of conduction electrons^15,17^. The significant decrease of absorption with decreasing temperature at energies such as 0.627 eV (corresponding to the laser line wavelength of 1.977 μm used for the magneto-transmission experiment) directly reflects the MI transition. The observed temperature dependence of the spectra is very similar to the previously reported result for VO_2_^15^ and V_1−*x*_W_*x*_O_2_ (*x* = 0.05)^17^. Because the lowest photon energy in this work is 0.5 eV, the onset of the absorption corresponding to the energy gap is not observed. According to previous work^17^, the energy gap for *x* = 0.06 is expected to be ~0.1 eV.

**Fig. 2:**
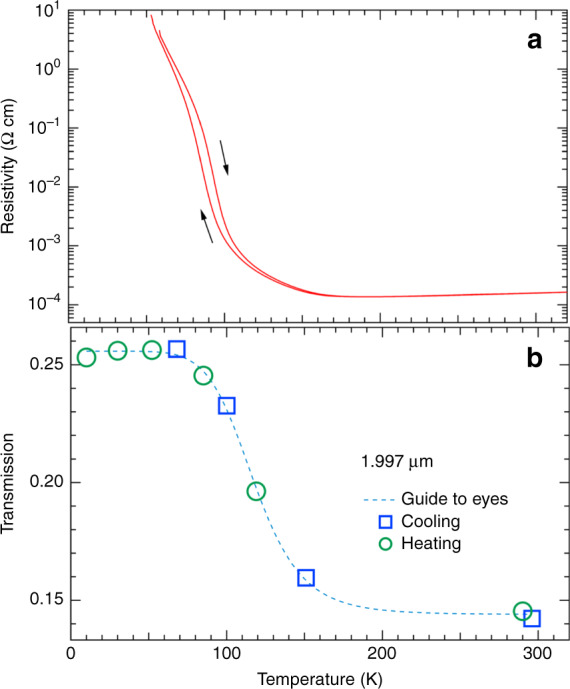
Temperature dependence of electrical resistivity and optical transmission of a V_1−*x*_W_*x*_O_2_ (*x* = 0.06) thin film. **a** Temperature dependence of electrical resistivity (*ρ*). The arrows indicate heating and cooling processes. **b** Temperature dependence of the optical transmission at 1.977 μm. The transmission measured in cooling process and that in heating process are represented with blue open squares and green open circles, respectively. The error bar is as large as the size of the square and circle marks in the plot.

  Figure 2a and b show, respectively, the temperature dependence of electrical resistivity (*ρ*) and that of the optical transmission at 1.977 μm of three layers of a V_1−*x*_W_*x*_O_2_ (*x* = 0.06) thin film with a total thickness of 45 nm.

The MI transition occurs at ~100 K, and the transmission below ~70 K is nearly independent of temperature. The curve-fitting analysis of the spectra, taking into account the DC electrical resistivity, shows that the effect of the closing of the energy gap (change in the absorption band) is more significant than that of the free-carrier absorption. When the carrier density is less than ~10^25^ m^−3^, the transmission at 0.627 eV is nearly independent of the carrier density. Details of the fitting are described in the Supplementary Note 3. The hysteresis observed in the temperature dependence of *ρ* indicates the first-order nature of the MI transition, although it is not observed very clearly in the temperature dependence of the transmission. The transmission is measured with both cooling and heating processes, and Fig. 2b plots results of the cooling and heating processes with blue open squares and green open circles, respectively.

**Fig. 3:**
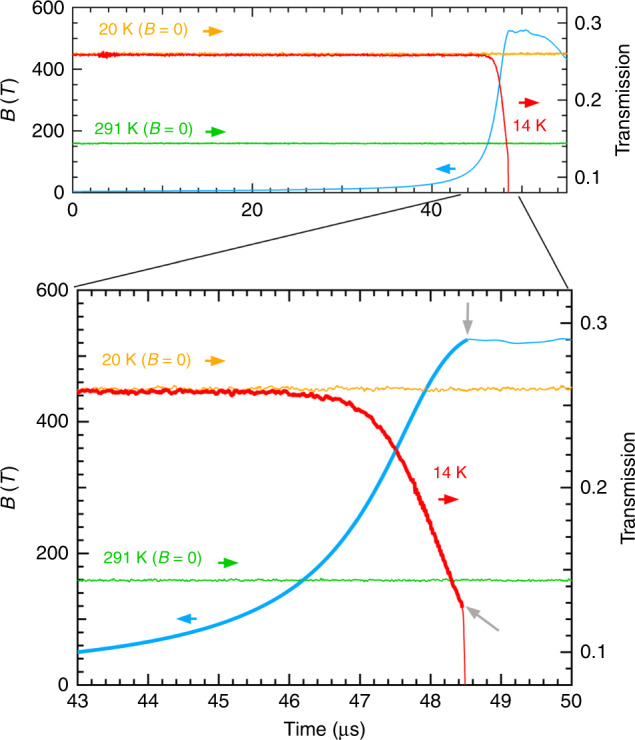
Temporal evolutions of the magnetic field and optical transmissions. Upper panel: evolutions of the magnetic field (blue curve) and the optical transmission at 14 K (red curve). The temporal evolutions of the transmission at 20 K and 291 K before applying the magnetic field are also shown with orange and light green curves, respectively. Lower panel: magnified view of the upper panel. The two grey arrows show the breaking points of the measurements of the magnetic field and optical transmission, respectively. They are found to occur at nearly the same time as the timing of termination of the magnetic-flux compression process^23^. The magnetic field and optical transmission at 14 K after the breaking points are denoted with thin curves.

Ultra-high magnetic fields (*B*) of up to 520 T are applied perpendicular to the thin-film plane (*B*∣∣*c*, where *c* is the crystal axis of the rutile structure), and the optical transmission at 1.977 μm is simultaneously measured. As shown in the upper panel of Fig. 3, the magnetic field (blue curve) increases with time (*t*) and reaches 520 T at 48.4 μs after the ignition of the gap switches of the capacitor bank power supply at *t* = 0. Figure 3 shows the *t* evolution of the transmitted light intensity at 14 K with a red curve. The lower panel of the figure shows a magnified view of the region from 50 to 520 T. The optical transmission at 20 and 291 K without magnetic fields is measured immediately before conducting the destructive ultra-high magnetic-field experiment, clearly showing the transmission levels in the insulating and metallic phases, respectively.

The transmission at 14 K under a magnetic field starts to show a gradual decrease at ~45 μs when the magnetic field reaches ~100 T; subsequently, the decrease is accelerated with increasing magnetic field. Then eventually, the transmission under fields >500 T is less than that in the high-temperature metallic phase (291 K), which clearly shows that the insulating phase of V_1−*x*_W_*x*_O_2_ (*x* = 0.06) is transformed to a metallic phase under ultra-high magnetic fields exceeding 500 T. Because the transmission at 14 K keeps decreasing beyond the value corresponding to the high-temperature metallic phase, the electrical resistivity is expected to be lower in the high-field metallic phase than in the high-temperature metallic phase. A lower scattering rate of electrons is expected owing to reductions in phonon, and magnetic scattering in the low-temperature high magnetic-field metallic phase.

**Fig. 4:**
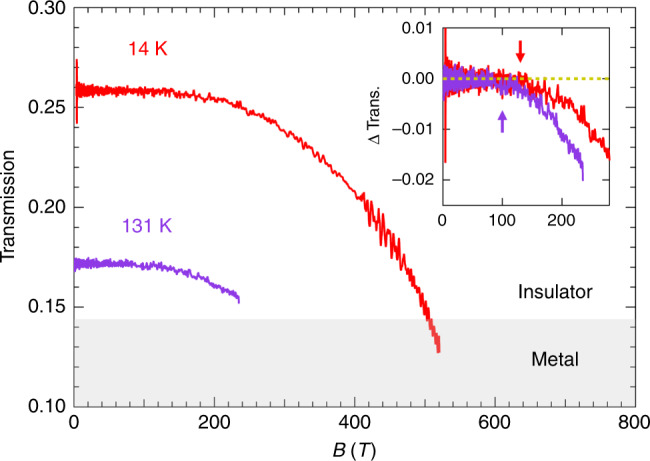
Magnetic-field dependence of the optical transmission at 14 and 131 K. The transmission is plotted before the breaking point of the destructive measurement. The shaded area indicates transmission lower than that in the high-temperature metallic phase at 291 K. Inset: transmission change ΔTrans. as a function of the magnetic field. The dashed yellow line corresponds to ΔTrans. =  0. The red and purple arrows indicate the field positions (B^*^) where ΔTrans. deviates from zero.

  Figure 4 plots the transmission at 14 and 131 K as functions of the magnetic field. Because the experiment at 131 K was performed with a lower energy for magnetic-field generation, the maximum field was 240 T. The transmission at zero field is lower than that at 14 K because the temperature is close to *T*_MI_. The inset shows the change in the transmission (Δ Trans.) as a function of the magnetic field. Δ Trans. is found to become finite at a certain magnetic field (B^*^), as indicated by the arrow for each temperature. B^*^ is evaluated as ~120 and 100 T at 14 and 131 K, respectively. The considerable decrease of optical transmission at 1.977 μm under an ultra-high magnetic field, as shown in Fig. 4, constitutes direct evidence of a magnetic-field-induced IM (MFI–IM) transition. Because the transmission change is induced at a threshold magnetic field B^*^, the MFI–IM transition is likely a first-order transition.

Here, we note that the transition is much broader than expected for a first-order transition. It may be because the inhomogeneous distribution of W sites^18^ creates several nanoscale domains with slightly different potential barriers. Thus, it is worth mentioning how the W doping affects the basic properties of VO_2_. As shown for the V_1−*x*_W_*x*_O_2_ (*x* = 0.06) thin film investigated in this work, doping the V sublattice with W reduces the MI transition temperature *T*_MI_ by  ~20 K/at.%W for the bulk^19^ and by  ~50 K/at.%W for nanostructures^13,20^. The microscopic origin of the reduced *T*_MI_ was revealed by the extended X-ray absorption fine structure as follows. The local lattice structure at W sites is more symmetric than that at V sites, and it induces the detwisting of the nearby asymmetric monoclinic VO_2_ lattice towards the rutile phase^18^. The W sites form rutile-like VO_2_ nuclei, and the propagation of these nuclei decreases the energy barrier of the phase transition^18^. Hence, the intrinsic mechanism of the MI transition of W-doped VO_2_ is identical to that of non-doped VO_2_. The broadening of the transition found in the temperature dependence of the electrical resistivity is considered to be due to the inhomogeneous distribution of W sites and reflects the growing so-called metallic puddles in the insulating host^18,21,22^. In addition, the growing part of the metallic puddles gradually reduces the optical transmission.

### V_1−*x*_W_*x*_O_2_ (*x* = 0, 0.036)

**Fig. 5:**
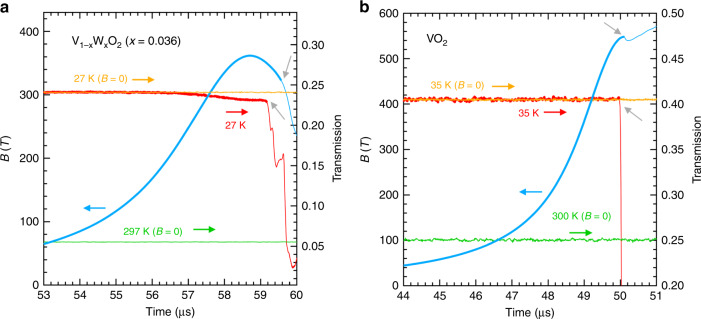
Temporal evolutions of the magnetic field and optical transmissions in V_1−*x*_W_*x*_O_2_. **a** Evolutions of the magnetic field (blue curve) and the optical transmission in V_1−*x*_W_*x*_O_2_ (*x* = 0.036) at 27 K (red curve). The temporal evolutions of the transmission at 27 K and 297 K before applying the magnetic field are also shown with orange and light green curves, respectively. The two grey arrows show the breaking points of the measurements of the magnetic field and optical transmission, respectively. The magnetic field and optical transmission in the field after the breaking points are denoted with thin curves. **b** Evolutions of the magnetic field (blue curve) and the optical transmission in VO_2_ at 35 K (red curve). The temporal evolutions of the transmission at 35 K and 300 K before applying the magnetic field are also shown with orange and light green curves, respectively. The two grey arrows show the breaking points of the measurements of the magnetic field and optical transmission, respectively. The magnetic field and optical transmission in the field after the breaking points are denoted with thin curves.

One of the most important experiments to confirm the validity of the discovered MFI–IM transition in V_1−*x*_W_*x*_O_2_ (*x* = 0.06) is to investigate the dependence of the transition on the W content (*x*). Figure 5a and b shows 1.977 μm magneto-transmission of other V_1−*x*_W_*x*_O_2_ thin films of *x* = 0.036 and 0, respectively. Three layers are stacked for the measurement of each *x*, as done in the measurement of the *x* =  0.06 films. The thicknesses of the films are 19 × 3 = 57 nm for *x* = 0.036 and 13 × 3 = 39 nm for *x* = 0. For the *x* = 0.036 films, it is found in Fig. 5a that a decrease of the transmission begins at ~56.5 μs when the magnetic field reaches 200 T. The highest magnetic field of this experiment is 360 T, which is relatively low because of the unexpected operation failure of the part of the ignition switches of the field generator. The decrease of the transmission indicates the start of metallisation, which is a distinct sign of the occurrence of the MFI–IM transition. The threshold magnetic field B^*^ in *x* = 0.036 is roughly 200 T, which is higher than B^*^ = 120 T for *x* = 0.06 at 14 K. Because the *T*_MI_ is ~100 K for *x* = 0.06 and that is ~195 K for *x* = 0.036, the higher B^*^ in *x* = 0.036 strongly indicates that the phenomenon of the MFI–IM transition can be understood in terms of the energy scale that determines the stability of the insulating phase.

Moreover, as shown in Fig. 5b, *x* = 0 (non-doped VO_2_) exhibits no change in the optical transmission with increasing magnetic fields up to 540 T. Because the *T*_MI_ of the VO_2_ film utilised is ~295 K, which is higher than those of *x* = 0.036 and 0.06, it is possible that the MFI–IM transition occurs at a magnetic field exceeding 540 T. More detailed characteristics of the films utilised are summarised in the Supplementary Note 1.

### Responses to the ultra-high magnetic field in V_1−*x*_W_*x*_O_2_ thin films

**Fig. 6:**
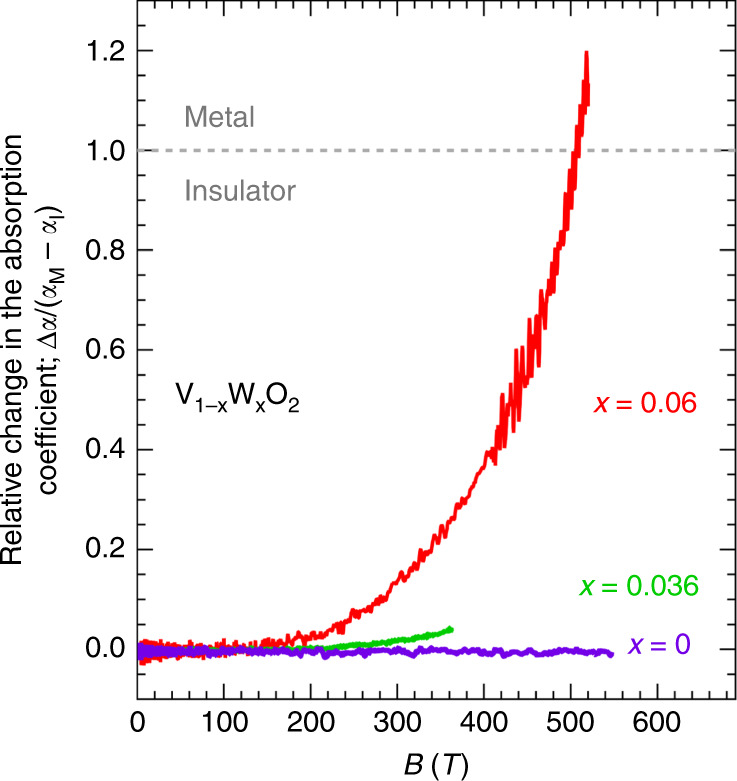
Relative change in the absorption coefficient (*α*) at 1.977 μm in V_1−*x*_W_*x*_O_2_ (*x* = 0, 0.036, 0.06). Δ*α*/(*α*_*M*_ − *α*_*I*_) is plotted as a function of magnetic field. *α*_*M*_ and *α*_*I*_ are defined as the *α* in the metallic and insulating phases at zero fields, respectively. Δ*α* is the magnetic-field-induced change in the *α*. The ratio of the measurement temperature *T* to *T*_MI_ for each film is 35/295  ≈  0.12 for VO_2_, 27/195  ≈  0.14 for *x* = 0.036, and 14/100 = 0.14 for *x* = 0.06.

  Figure 6 plots the relative change in the absorption coefficient (*α*) at 1.977 μm, Δ*α*/(*α*_*M*_ − *α*_*I*_), of the each film as a function of the magnetic field. *α*_*M*_ and *α*_*I*_ are defined as the *α* in the metallic and insulating phases at zero fields, respectively. Δ*α* is the magnetic-field-induced change in the *α*. Because the onset magnetic field B^*^ is higher in *x* = 0.036 than that in *x* = 0.06 by roughly 100 T, we may expect the metallisation for *x* = 0.036 at ~600 T, assuming a magnetic field variation of Δ*α*/(*α*_*M*_ − *α*_*I*_) similar to that for *x* = 0.06. A potential MFI–IM transition in the non-doped VO_2_ thin film is expected to be observed at fields exceeding 540 T. A 1000-T-class fields^23^ is necessary to resolve this intriguing problem.

### Discussion

**Fig. 7:**
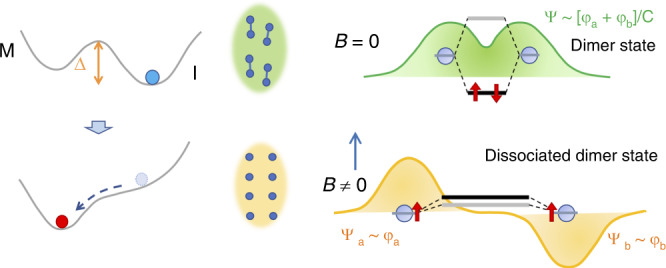
Schematic of the magnetic-field-induced insulator–metal (MFI–IM) transition. The left side shows that the potential barrier Δ is lowered because of the Zeeman energy. The middle part schematically shows the collapse of the V-V dimers. The right part shows that applying a magnetic field induces the dissociation of the dimer owing to the destabilisation of formation of the bonding state Ψ of the molecular orbital, where *φ*_*a*_ and *φ*_*b*_ are the wave functions of the independent vanadium ions.

It can be said that it is experimentally clear that W-doped VO_2_ transforms into a metallic phase in ultra-high magnetic fields and the transition magnetic field depends on *T*_MI_, which is a measure of the stability of the insulating phase. As schematically shown in Fig. 7, one of the most plausible explanations for the observed MFI–IM transition is that the V–V dimers are dissociated by the collapse of the molecular orbital. Because the molecular orbital is stable owing to the occupation of two electrons in the bonding state, and their spins need to be antiparallel, the spins of the two electrons parallel can disturb the formation of bonding between adjacent V atoms. The spin quantum number of V^4+^ (*d*^1^) is *S* = 1/2, and its Zeeman energy at 120 T corresponds to 162 K, which is close to the onset temperature for the MI transition in V_1−*x*_W_*x*_O_2_ (*x* = 0.06) at zero magnetic field, as shown in Fig. 2. This fact supports the interpretation above. The potential barrier Δ in Fig. 7 for the MFI–IM transition is expected to be as large as the Zeeman energy at ~120 T (200 T) for *x* = 0.06 (0.036), and it is larger than the Zeeman energy at 500 T for non-doped VO_2_. In contrast, the energy separation of the bonding and anti-bonding orbitals of the V–V dimer is expected to be ~2.5 eV^6,24^. Because this energy scale corresponds to 30,000 K, which is more than two orders of magnitude larger than the Zeeman energy, the observed magnetic-field-induced metallisation cannot be explained by considering only an isolated single dimer. Some many-body interactions through the electron correlation^4,10,25^ might be necessary to understand the mechanism of dissociation of the dimers by controlling the electron spins.

On the other hand, from the technical point of view, one may wonder how the sample temperature remains stable during the application of pulsed magnetic fields. When the sample is electrically conducting, eddy currents can heat it up. Because the pulse duration of the magnetic field in this work is of the microsecond order, the time derivative of the magnetic field *dB*/*dt* is large and may induce a significant increase in the sample temperature. We evaluated the temperature rise of the sample when it was in the insulating phase and found that the temperature rise was less than 4 K even at 500 T for *x* = 0.06. We also found that even after the MFI–IM transition occurs and the resistivity becomes low, the temperature cannot increase thanks to a very rapid thermal relaxation to the TiO_2_ substrate. It happens within several tens of picoseconds because of the very small thickness of the sample (e.g., 15 nm for *x* = 0.06). Actually, maintaining an isothermal condition during magnetic field sweeping in the microsecond time range has been experimentally confirmed using a sinusoidal-like waveform of a pulsed magnetic field, in which the *dB*/*dt* reaches the maximum at the starting point of the field. As shown in the Supplementary Fig. 5, the optical transmission of *x* = 0.06 films is measured at 95 K with such a sinusoidal-like-waveform magnetic field of up to 140 T. (The field is generated by the single-turn-coil technique^26^.) According to the calculation without taking into account the thermal relaxation to the substrate, the temperature quickly increases immediately after applying the magnetic field and reaches 300 K at 10 T. Hence, a large decrease of the transmission due to the metallisation should be observed immediately after applying the pulsed field. However, actually, the measured optical transmission is found to be almost constant up to ~100 T and even exhibits a slight decrease at fields exceeding 100 T, which is in good agreement with our observation in the 500-T experiment. This experimental finding proves that a significant temperature rise never happens even in a microsecond ultra-high magnetic field, and the rapid thermal relaxation maintains the isothermal condition. A detailed discussion and evaluation of the sample temperature are presented in the Supplementary Note 2.

In summary, we demonstrated that an ultra-high magnetic field of 500 T can transform the insulating dimerised state of W-doped VO_2_ to a metallic state at low temperatures. We showed that the V–V dimers are dissociated by the collapse of the molecular orbital; the bonding state becomes unstable with the aligning of electron spins in the magnetic-field direction. The *d* electrons participating in the metal–metal bonding become itinerant after the breaking of the dimers. This phenomenon is similar to chemical catastrophe: the chemical bonding is collapsed by a very strong magnetic field through the spin Zeeman effect^27,28^. It had been considered to occur only in cosmic spaces such as on a neutron star, where a very strong magnetic field exceeding 10^6^ T exists. This work suggests that the formation of the molecular orbital between the vanadium ions, which results in the localisation of the unpaired *d* electrons, is the predominant driving force behind the MI transition. On the other hand, the Zeeman energy corresponding to 500 T is ~60 meV, which is less than the eV-order binding energy for a local isolated V–V dimer by more than two orders of magnitude. Therefore, it is likely that the electron correlation must also be included to obtain a quantitative understanding of the MFI–IM transition^4,10,25^.

From the perspective of material science, because a singlet spin state with the formation of a cluster of magnetic atoms is exhibited by various other strongly correlated insulators, such as Ti_2_O_3_^29,30^, AlV_2_O_4_^31^ and CuIr_2_S_4_^32^, investigation of the effect of a magnetic field on their electronic states is an intriguing and important research problem. A magnetic field of the order of 1000 T is indispensable for such research because of their high *T*_MI_ of several hundreds of kelvins^1^.

## Methods

### Preparation of the films

Thin films of V_1−*x*_W_*x*_O_2_ (*x* = 0, 0.036, 0.06) were prepared using pulsed laser deposition on TiO_2_ (001) substrates^33^. The detailed characteristics of the thin films are described in the Supplementary Note 1.

### Characterisation of the films and magneto-transmission measurements

The optical absorption spectra at zero field and different temperatures are acquired using a commercial spectrometer (JASCO V570) in the transmission configuration. The temperature dependence of the electrical resistivity was measured using the conventional four-probe method. Magneto-transmission measurements were performed using an infrared fibre laser (AdValue Photonics: AP-TM-1975-SM-05) having a wavelength of 1.977 μm. A HgCdTe photodiode was used for detecting the intensity of the transmitted light. A 1000-T field generator using electromagnetic flux compression^23^ was employed to obtain a strong magnetic field of up to 540 T. The infrared laser and HgCdTe detector were placed in a shield room to avoid electromagnetic noise during the magnetic-field generation. The sample has an area of 1.8 × 1.8 ×  mm^2^, and it is sandwiched by two optical fibres having a core diameter of 800 μm, which are used for relaying incoming and transmitted laser light. Helium-flow-type cryostats made of plastic were used to achieve low temperatures for the magneto-transmission experiments under ultra-high magnetic fields.

## Supplementary information

Supplementary Information

## Data Availability

All relevant data in this paper are available from the authors upon reasonable request. The authors think that the request-based data sharing would be reasonable and effective.
