# Supplementary material for: Magnetic-field-induced insulator–metal transition in W-doped VO2 at 500 T
Source: Nat Commun. 2020 Jul 17;11:3591. doi: 10.1038/s41467-020-17416-w (PMC7367819; doi:10.1038/s41467-020-17416-w)
Supplement: Supplementary file 1 — Supplementary Information [file 41467_2020_17416_MOESM1_ESM.pdf]

Supplementary Information :

Magnetic-field-induced insulator-metal transition in W-doped VO_2 at 500 T

Matsuda *et al.*

Supplementary Note 1. Characterization of the W-doped VO_2 thin films

$V_{1-x}W_xO_2$ (x=0, 0.036, 0.06) thin films were grown on TiO_2 (001) substrates with a pulsed laser deposition technique [1, 2]. The XRD pattern of each film is shown in Figs. 1 (a) and (b). A Cu-Kα line was utilised for the measurements. The 002 peak of the $V_{1-x}W_xO_2$ film and that of the TiO_2 substrate are observed in Fig.1 (a). Only the 002 peaks of the $V_{1-x}W_xO_2$ film were also measured with higher signal-to-noise ratio (Fig.1 (b)). It has been found that they shift to lower angle with increasing x, which indicates an elongation of the c axis of the rutile structure [2]. We have evaluated the x from the lattice constant of the c axis and the error of the estimated x is $\pm$0.005. Because no other peak is observed, the each film is considered to be an (001)-oriented single phase. The electrical resistivities of the films are shown as a function of temperature in Fig.1 (c). A clear metal-insulator (MI) transition is observed in each film as a steep rise of the resistivity. The transition temperatures are roughly 100, 200, and 300 K for x =0.06, 0.036, 0, respectively, with the hysteresis temperature width of 5 – 7 K. The observed temperature dependence of the electrical resistivity and its x variation are similar with the results reported in the previous study [2].

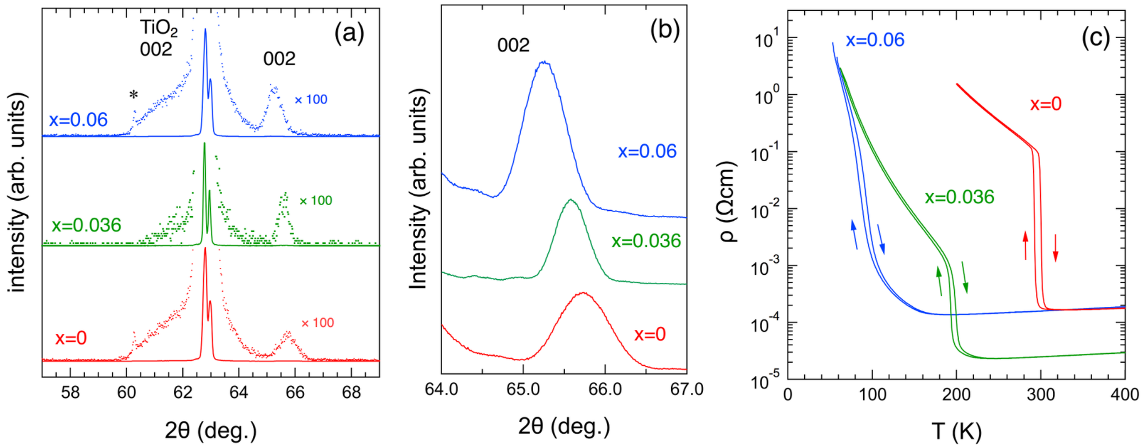

Supplementary Figure 1. (a) X-ray diffraction (XRD) patterns of the $V_{1-x}W_xO_2$ (x =0, 0.036, 0.06) thin films measured in the present study. The film thicknesses are 13, 19, and 15 nm for x = 0, 0.036, and 0.06, respectively. The pattern magnified 100 times is also shown with a dotted curve for each film. The patterns are vertically shifted for clarity. * : contamination originating from the XRD apparatus used. (b) The XRD patterns of the $V_{1-x}W_xO_2$ (x =0, 0.036, 0.06) thin films measured with higher X-ray power. (c) The temperature dependence of the electrical resistivities of the $V_{1-x}W_xO_2$ thin films. A hysteresis is seen in the temperature variation, which indicates that the MI transition is first order. Arrows denote the temperature increasing and decreasing processes.

Figure 2(a) shows the optical absorption spectra in the $V_{1-x}W_xO_2$ (x =0.036) thin film at different temperatures. The dashed line indicates the energy position of the 1.977 μm laser line (0.627 eV) at which the magneto-transmission experiment is conducted. A strong absorption at low energy region at high temperatures and reduction of it with decreasing temperature are similarly observed with that in the x = 0.06 film shown in Fig. 1 in the main text. The temperature dependence of the optical transmission at 1.977 μm is shown in Fig.2(b). A distinct transmission increase is observed with decreasing temperature at approximately 195 K, which corresponds to the steep rise of the electrical resistivity in the x = 0.036 film as shown in Fig. 1(b).

Figure 3(a) shows the optical absorption spectra in the VO_2 thin film at different temperatures. The dashed line indicates the energy position of the 1.977 μm laser line (0.627 eV) at which the magneto-transmission experiment is conducted. The behavior of the temperature variation of the absorption spectrum is similar to those observed in x = 0.06 and 0.036 films, and clearly shows the MI transition. The temperature dependence of the optical transmission at 1.977 μm is shown in Fig.3(b). Although a hysteretic behavior is successfully observed in the change of the optical transmission, the quantitative analysis is difficult because the amount of the hysteresis is comparable to the error bar of the measurement.

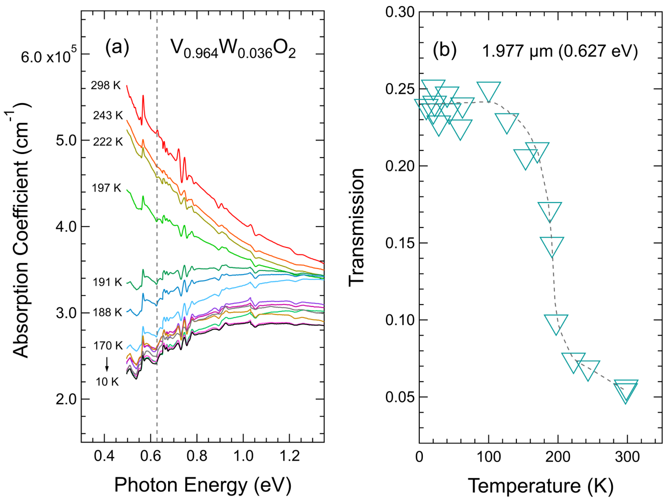

Supplementary Figure 2. (a) The absorption spectra in the $V_{1-x}W_xO_2$ (x= 0.036) thin film in a near infrared region at different temperatures. The measurement was made in temperature decreasing process. The dashed vertical line corresponds to the photon energy of the 1.977 μm (0.627 eV) laser line. (b) The temperature dependence of the optical transmission of the three films of $x = 0.036$ (the total thickness is 57 nm) at 0.627 eV. The grey dashed curve is a guide for eyes.

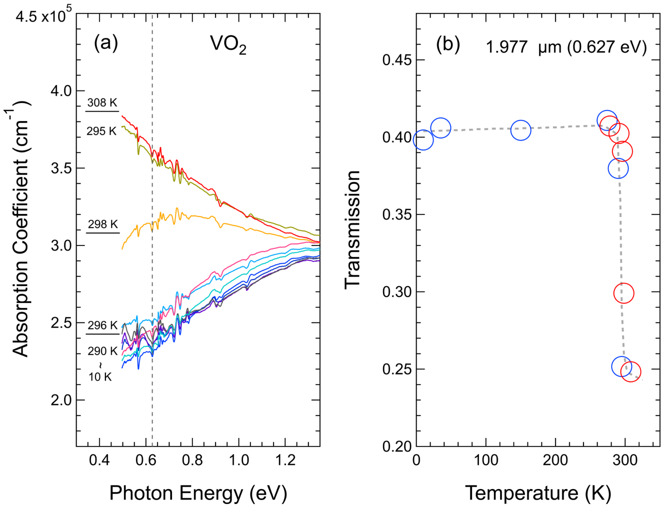

Supplementary Figure 3. (a) The absorption spectra in the VO_2 thin film in a near infrared region at different temperatures. The dashed vertical line corresponds to the photon energy of the 1.977 μm (0.627 eV) laser line. The temperatures with under lines at high temperatures indicate that the measurements at these temperatures were done in the temperature increasing process. (b) The temperature dependence of the optical transmission of three films of VO_2 (the total thickness is 39 nm) at 0.627 eV. The blue and red open circles denote the results in the temperature decreasing and that in the temperature increasing process, respectively. The grey dashed curve is a guide for eyes.

Supplementary Note 2. Evaluation of heating of the sample in pulsed magnetic fields

When pulsed magnetic fields are applied to an electrically conducting sample, the sample temperature (T) can change with magnetic field (B) due to the eddy current heating. Here we evaluate the temperature rise (ΔT) of a

$V_{1-x}W_xO_2$ ($x = 0.06$) thin film in ultrahigh pulsed magnetic fields. The ΔT is calculated as follows,

$$\Delta T = \frac{x^2}{8}\int_0^t \frac{1}{\rho c_V}\left(\frac{dB}{dt}\right)^2 dt \tag{1}$$

,where x, c_V, and ρ are the radius, specific heat, and electrical resistivity of the sample, respectively. The calculated ΔT at different measurement conditions are shown in Figs. 4 (a), (b), and (c). The x is 0.9 mm and c_V is assumed to be βT^3, where $\beta \sim 7.4 \times 10^{-2}$ J m^{-3} K^{-4} is obtained from c_V =2 MJ m^{-3} K^{-1} at 300 K for VO_2 [3]. The ρ and c_V used are shown in Table 1.

Supplementary Table 1. Parameters use for calculation of the ΔT

	14 K	95 K	131 K
ρ [Ωm]	10	6 $\times 10^{-5}$	3×10^{-6}
c_V [J m^{-3} K^{-1}]	200	6 $\times 10^4$	2×10^5

As shown in Fig. 4 (a), the ΔT is found to be smaller than 4 K if the initial temperature $T_{\rm ini}$ = 14 K even at 500 T. Hence the significant change in the optical transmission observed in high magnetic fields exceeding 120 T when $T_{\rm ini}$ = 14 K cannot be attributed to the effect of the eddy current heating.

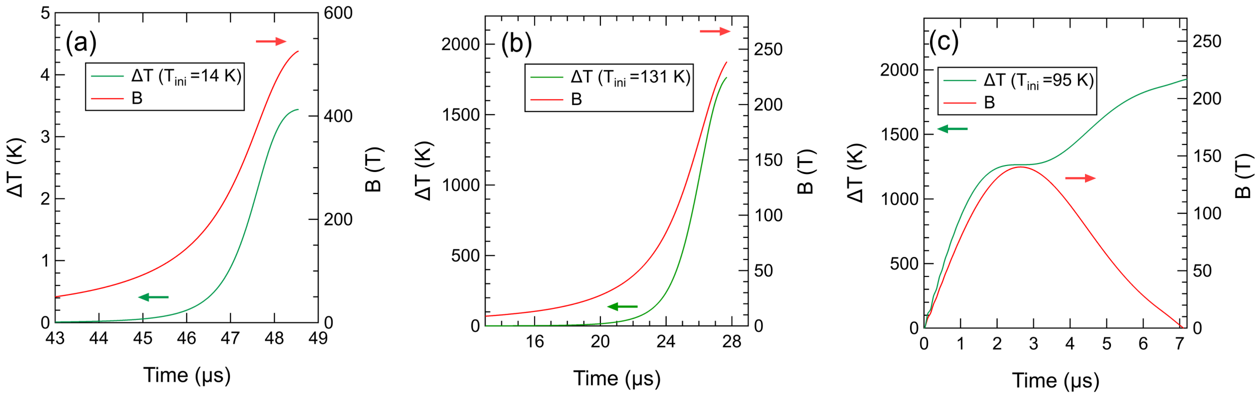

Supplementary Figure 4. Evolution of the temperature rise ΔT in the $V_{1-x}W_xO_2$ ($x = 0.06$) thin film and the B curve as a function of time. (a) The initial temperature $T_{\rm ini}$ = 14 K and the B is generated by the electromagnetic-flux-compression [4]. (b) $T_{\rm ini}$ = 131 K and B is generated by the electromagnetic-flux-compression. (c) $T_{\rm ini}$ = 95 K and B is generated by the single-turn coil technique [5].

On the other hand, the calculated ΔT for $T_{\rm ini}$ = 131 K in B of up to 240 T (Fig. 4 (b)) suggests that significant heating of the sample takes place. It is because the ρ is rather small reflecting metallic nature. The calculated ΔT reaches 100 K even at a low field of around 60 T. However, the experimentally obtained optical transmission is found to show nearly no B dependence when the field is lower than 100 T, which indicates that the actual ΔT is smaller than a few Kelvin.

The similar finding is obtained from another experiment using the single-turn coil technique [5]. The waveform of B is different from the one obtained by electromagnetic flux compression. As shown in Fig. 4 (c), the sinusoidal like B curve can induce the eddy current heating just after application of field. The corresponding optical transmission experiment was conducted on the $V_{1-x}W_xO_2$ ($x = 0.06$) thin film at 95 K. Fig. 5 (a) shows the time evolution of B and that of transmission at 1.977 μm. A significant decrease of the transmission is expected from the calculated ΔT because the temperature of the sample becomes 300 K at 10 T. One find, however, that the transmission keeps the initial value up to around 100 T. Moreover, a slight decrease of the transmission observed at field exceeding 100 T (Fig. 5 (b)) agrees with the results of higher field experiments up to 240 and 520 T.

From the experimental findings that transmission at 95 K and 131 K seem to be free from eddy-current heating, we can conclude that the calculated ΔT with adiabatic condition shown in Fig. 4 considerably overestimate the effect of sample heating. It should be taken into account transferring heat from the sample to the surrounding heat bath. In the present study, the heat generated in the sample actually can diffuse to the TiO_2 substrate and the heat transfer can be very fast because the thickness of the $V_{1-x}W_xO_2$ ($x = 0.06$) film (d) is only 15 nm. Insulating and nonmagnetic TiO_2 substrate of which thickness is 0.5 mm would work as an ideal thermal bath.

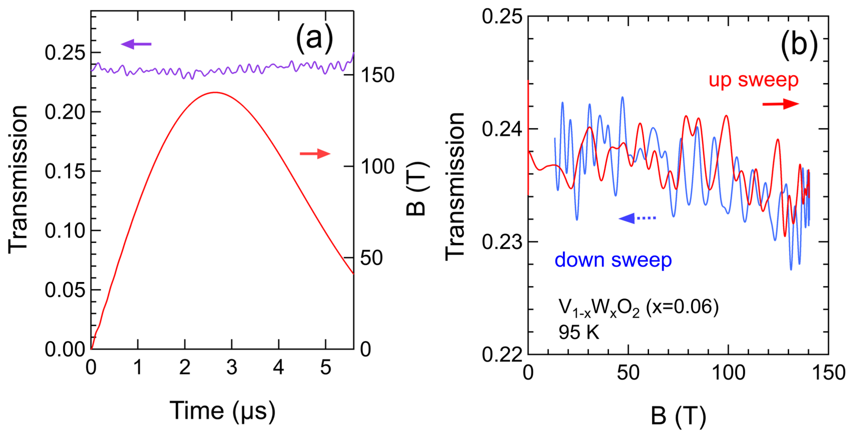

Supplementary Figure 5. (a) Time dependence of B and that of the optical transmission of the $V_{1-x}W_xO_2$ ($x = 0.06$) thin film at 1.977 μm. (b) Plot of the optical transmission as a function of B.

The speed of the temperature transportation of the sample with the surface area A and volume V can be evaluated as follows using the Fourier's law,

$$\frac{\partial T}{\partial t} = -\left(\frac{k}{c_V}\right)\frac{A}{V}\frac{\partial T}{\partial x} \tag{2}$$

, where k is the thermal conductivity. The speed $\partial T/\partial t$ is proportional to the gradient of the temperature in space $\partial T/\partial x$. Here $A/V = d = 15$ nm, and k can be taken to be 20 W m^{-1} K^{-1} for metallic VO_2 [3].

To obtain isothermal condition for measurements, it is required to obtain thermal equilibrium condition by fast heat exchange with surrounding thermal bath. Considering $\Delta T = 1$ K at the surface of the film, the distance between the surface and the substrate of 15 nm gives the relation

$$\frac{\partial T}{\partial x} \sim \frac{\Delta T}{\Delta x} = \frac{1 \text{ K}}{15 \times 10^{-9}\text{m}} \sim 6.7 \times 10^{7} \text{ K m}^{-1}. \tag{3}$$

Then we have,

$$\frac{\partial T}{\partial t} \sim -\frac{20}{2 \times 10^{6}}\left(\frac{1}{15 \times 10^{-9}}\right)(6.7 \times 10^{7}) \sim 4.5 \times 10^{10} \text{ K s}^{-1}. \tag{4}$$

This is the speed of temperature transfer. Temperature increase of 1 K at the surface can be transferred to the interface between the sample and the TiO_2 substrate in $(4.5 \times 10^{10})^{-1}$ s $\sim 2.2 \times 10^{-11}$ s = 22 ps. This time scale is six orders of magnitude smaller than the duration time of the magnetic field and thus the isothermal condition is expected to be maintained during the B pulse. This fast thermal relaxation can explain the experimental findings that the isothermal condition is likely to be maintained during the microsecond ultrahigh B pulse.

Supplementary Note 3. Curve fitting of the absorption spectra at different temperatures

The optical absorption spectra of the $V_{1-x}W_xO_2$ ($x = 0.06$) thin film are analyzed. In the insulating phase, as shown in Fig. 6 (a), the spectrum exhibits a clear absorption band around 1 eV and another absorption rise starts at around 2 eV indicating larger absorption band at higher energy. According to the previous studies [6–8], they are the absorption bands due to the $d_{||} \to \pi^*$ and $d_{||} \to \sigma^*$ transitions, respectively. Here $d_{||}$ is the bonding orbital of the vanadium dimers and π^* and σ^* are the orbitals originate from t_{2g} and e_g orbitals, respectively. Because the σ^* is rather strongly hybridized with oxygen $2p$ orbital, the latter transition can be regarded as a charge transfer (CT) absorption. On the other hand, π^* has mostly d_{xz} and d_{yz} character of d electrons of a vanadium atom [7]. A lognormal function is used for representing the $d_{||} \to \pi^*$ transition contribution since the peak shape is asymmetric [2, 9], while a Gauss function is used to fit the slope of the the CT transition.

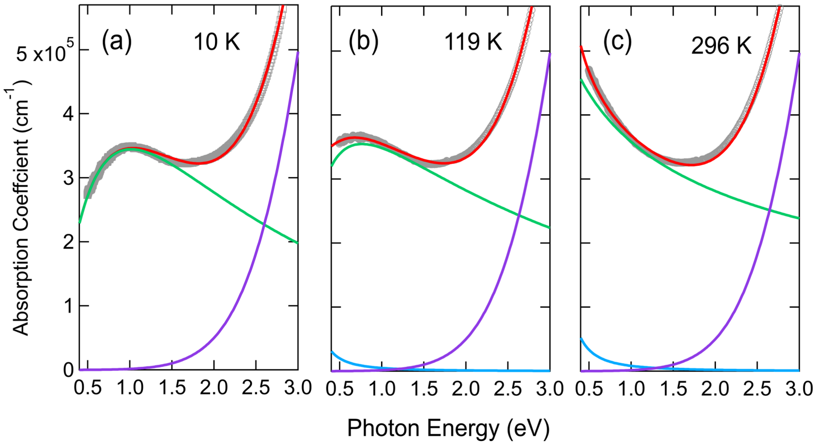

Supplementary Figure 6. Results of the curve fitting (thick solid curves) for the optical absorption spectra of the $V_{1-x}W_xO_2$ ($x = 0.06$) thin film at different temperatures. The green and purple curves correspond to the $d_{||} \rightarrow \pi^*$ and the CT transitions, respectively. The light blue curve is the Drude component, and the red curve contains the total absorption components. The experimental results are represented by grey open marks. (a) 10 K. (b) 119 K. (c) 296 K.

In addition to the two absorption bands, free carrier absorption (so-called Drude absorption) is taken into account for the spectra fitting. The absorption coefficient α for the Drude component is expressed as follows.

$$\alpha = \frac{\omega_p^2 \lambda^2}{4\pi c^3 \bar{n} \tau}. \tag{5}$$

Here, the plasma frequency ω_p is proportional to the square root of the carrier density n_e.

$$\omega_p = \sqrt{\frac{n_e e^2}{\varepsilon_0 m^*}}. \tag{6}$$

λ is the wavelength, c is the speed of light, $\bar{n}$ is the reflective index, and τ is the scattering time. e is the electronic charge. ε_0 and m^* are the dielectric constant of vacuum and the effective mass, respectively. $m^* = 3m_0$ [9–11] and $\bar{n}$ =3 [12] are used for the fitting, where the m_0 is the free electron mass.

The representative optical absorption spectra at 10, 119, and 296 K are shown in Fig. 6 along with the fitting curves. The red curve is the result of the fitting. The green and purple curves are the components of $d_{||} \rightarrow \pi^*$ and the CT transitions, respectively. The peak energy of the Lognormal function (E_0) seems to change with temperature, suggesting significant change in the electronic structure due to the metal-insulator transition of this sample around 100 K.

The Drude term is not significant at temperatures lower than around 70 K at which the n_e is estimated to be around 10^{25} m^{-3}. At higher temperatures, the Drude term contributes the optical absorption (light blue curve in Fig. 6). Because there are a lot of adjustable parameters and the slope due to the CT transition seems to be rather independent of temperature, we assume that the CT transition does not depend on temperature. Regarding the Drude absorption, we tried to find n_e and τ that give a good fitting results and simultaneously explains the DC electrical resistivity shown in Fig. 7 (a). The DC electrical resistivity ρ is assumed to be expressed as follows.

$$\rho = \frac{m^*}{e^2 n_e \tau}. \tag{7}$$

The light blue open squares shown in Fig. 7 (a) are the evaluated ρ with the parameters used for the curve fitting for the optical absorption spectra. The parameters used are shown in Fig. 7 (b) as a function of temperature. Although the Drude theorem can be too simple to evaluate the electronic state of $V_{1-x}W_xO_2$ ($x = 0.06$), the obtained n_e at higher temperature around 10^{28} m^{-3} is in agreement with the expected carrier density 3.3×10^{28} m^{-3} assuming one electron per formula unit of VO_2. (Here we use the density 4.57 g cm^{-3} and effects of W-doping is not taken into account.) Because the scattering time of carriers in metallic VO_2 can be estimated as an order of $10^{-15} \sim 10^{-14}$ s^{-1} [9], the τ shown in Fig. 7 (b) also seem be rather reasonable values .

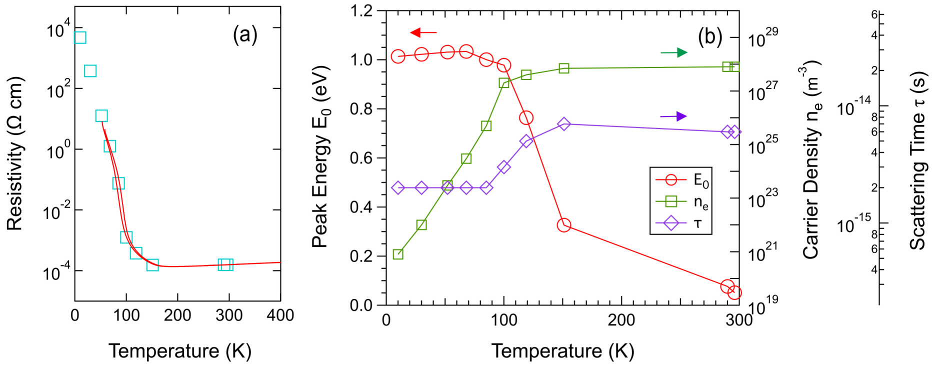

Supplementary Figure 7. (a) The DC electrical resistivity of the $V_{1-x}W_xO_2$ ($x = 0.06$) thin film as a function of temperature. (b) The deduced fitting parameters as a function of temperature; E_0 is the energy peak of the $d_{||} \rightarrow \pi^*$ transition, n_e is the carrier density, and the τ is the scattering time of the carrier.

As shown in the results of the curve fitting (Fig. 6), it is found that the contribution to the absorption at 0.627 eV (1.977 μm) in the spectra of $V_{1-x}W_xO_2$ ($x = 0.06$) mainly comes from the absorption due to the $d_{||} \rightarrow \pi^*$ transition with a small contribution of the free carrier absorption. Therefore, the observed significant decrease of the transmission at 1.977 μm in the ultrahigh magnetic fields exceeding 100 T to 520 T can be attributed to the change in the electronic state. The $d_{||} \rightarrow \pi^*$ absorption band shifts to the lower energy with magnetic field and most probably close the energy gap at around 500 T.

Supplementary References

[1] Y. Muraoka and Z. Hiroi, Metal-insulator transition of vo_2 thin films grown on tio_2 (001) and (110) substrates, Applied Physics Letters **80**, 583 (2002)

[2] K. Shibuya, M. Kawasaki, and Y. Tokura, Metal-insulator transition in epitaxial $v_{1-x}w_xo_2$ ($0 \leq x \leq 0.33$) thin films, Applied Physics Letters **96**, 022102 (2010)

[3] G. Hamaoui, N. Horny, C. Gomez-Heredia, J. Ramirez-Rincon, J. Ordonez-Miranda, C. Champeaux, F. Dumas-Bouchiat, J. Alvarado-Gil, Y. Ezzahri, J. K., and M. Chirtoc, Thermophysical characterisation of vo_2 thin films hysteresis and its application in thermal rectification, Sci. Rep. **9**, 8728 1 (2019)

[4] D. Nakamura, A. Ikeda, H. Sawabe, Y. H. Matsuda, and S. Takeyama, Record indoor magnetic field of 1200 t generated by electromagnetic flux-compression, Review of Scientific Instruments **89**, 095106 (2018).

[5] N. Miura, T. Osada, and S. Takeyama, Research in super-high pulsed magnetic fields at the megagauss laboratory of the university of tokyo, J. Low Temp. Phys **133**, 139 (2004)

[6] A. Gavini and C. C. Y. Kwan, Optical properties of semiconducting vo_2 films, Phys. Rev. B **5**, 3138 (1972)

[7] V. Eyert, The metal-insulator transitions of vo_2: A band theoretical approach, Annalen der Physik **11**, 650 (2002)

[8] H. He, A. X. Gray, P. Granitzka, J. W. Jeong, N. P. Aetukuri, R. Kukreja, L. Miao, S. A. Breitweiser, J. Wu, Y. B. Huang, P. Olalde-Velasco, J. Pelliciari, W. F. Schlotter, E. Arenholz, T. Schmitt, M. G. Samant, S. S. P. Parkin, H. A. Dürr, and L. A. Wray, Measurement of collective excitations in vo_2 by resonant inelastic x-ray scattering, Phys. Rev. B **94**, 161119 (2016)

[9] K. Okazaki, S. Sugai, Y. Muraoka, and Z. Hiroi, Role of electron-electron and electron-phonon interaction effects in the optical conductivity of vo_2, Phys. Rev. B **73**, 165116 (2006)

[10] W. H. Brito, M. C. O. Aguiar, K. Haule, and G. Kotliar, Metal-insulator transition in vo_2: A DFT + DMFT perspective, Phys. Rev. Lett. **117**, 056402 (2016)

[11] Y. Muraoka, H. Nagao, Y. Yao, T. Wakita, K. Terashima, T. Yokoya, H. Kumigashira, and M. Oshima, Fermi surface topology in a metallic phase of vo_2 thin films grown on tio2(001) substrates, Sci. Rep. **8**, 17906 (2018)

[12] H. W. Verleur, A. S. Barker, and C. N. Berglund, Optical properties of vo_2 between 0.25 and 5 ev, Phys. Rev. **172**, 788 (1968)
